# A phase one trial of carfilzomib, bendamustine, and dexamethasone in relapsed and/or refractory multiple myeloma

**DOI:** 10.1002/ajh.26178

**Published:** 2021-05-06

**Authors:** Hans C. Lee, Lei Feng, Onyeka Oriabure, Vivian Graham, Wendy Chen, Maria Badillo, Rebecca Lu, Hun J. Lee, Preetesh Jain, Elisabet E. Manasanch, Robert Z. Orlowski, Michael L. Wang

**Affiliations:** ^1^ Department of Lymphoma/Myeloma The University of Texas MD Anderson Cancer Center Houston Texas USA; ^2^ Department of Biostatistics The University of Texas MD Anderson Cancer Center Houston Texas USA

To the Editor:

The incorporation of novel agents including immunomodulatory drugs (IMiDs), proteasome inhibitors (PIs), and monoclonal antibodies (mAbs) to myeloma treatment regimens have led to substantial gains in overall survival in patients with multiple myeloma over the last 10–15 years. However, alkylating agents remain an important option in the myeloma therapeutic armamentarium, and their use in combination with novel agents have shown to be an effective treatment strategy for both newly diagnosed and relapsed and/or refractory myeloma patients.^1^

Bendamustine is an alkylating agent with a unique structure containing both a nitrogen mustard group and a benzimidazole ring. The latter may also confer antimetabolite properties to bendamustine, which is absent in other alkylator drugs commonly used in myeloma such as melphalan and cyclophosphamide.^2^ Moreover, mechanistic studies suggest that bendamustine induces more extensive and durable double‐stranded DNA breaks compared to other alkylator drugs, possibly through activation of a more complex base excision DNA repair pathway rather than the alkyltransferase DNA repair mechanism.^3^ The safety and efficacy of bendamustine have been demonstrated in combination with IMiDs^4^ and the first generation PI bortezomib.^5^ More recently, the safety and efficacy of bendamustine in combination with the irreversible second generation PI carfilzomib have been reported in newly diagnosed multiple myeloma patients.^6^ We herein report the results of a phase one investigator‐initiated study of carfilzomib, bendamustine, and dexamethasone in relapsed and/or refractory multiple myeloma patients (RRMM) (NCT02095834).

In this two‐part phase one study, RRMM patients with ≥1 prior line of therapy were enrolled. In part one, patients received carfilzomib 20/27 mg/m^2^ on days 1, 2, 8, 9, 15, and 16, bendamustine 50 mg/m^2^ on days 1 and 2, and dexamethasone 20 mg on days 1, 2, 8, 9, 15, 16, 22, and 23 on a 28‐day cycle at dose level one, with increasing doses of carfilzomib and bendamustine in a 3 + 3 dose‐escalation (Table S1). In part two of the study, enrollment of an additional 19 patients was planned at the maximum tolerated dose (MTD). During cycles 4–12, the dosing frequency of bendamustine decreased to day 1 only and dexamethasone to days 1, 2, 15, and 16. Starting cycle 13, the dose frequency of carfilzomib decreased to days 1, 2, 15, and 16 and dexamethasone to days 1 and 2 only. Prophylactic granulocyte colony‐stimulating factor was not mandated per protocol. The study was approved by the MD Anderson Cancer Center Institutional Review Board and conducted in accordance with Declaration of Helsinki.

The primary endpoint of the study was to determine the MTD of carfilzomib, bendamustine and dexamethasone with dose‐limiting toxicities (DLTs) assessed during the cycle one (28‐day) DLT‐evaluable period. Safety and disease evaluations were conducted prior to each cycle of therapy. Adverse events (AEs) were graded according to Common Terminology Criteria for Adverse Events (CTCAE) Version 4.03. Disease response and progression were assessed per International Myeloma Working Group consensus criteria. The Kaplan–Meier method was used to estimate time‐to‐event outcomes including progression‐free survival (PFS), duration of response (DOR), and overall survival (OS).

Between April 29, 2014 and October 14, 2016, 18 patients were screened for the study, and 17 patients were eligible for participation and treated on study. Baseline patient characteristics are summarized in Table S2. The median age was 63 years and median prior lines of therapy was four (range 1–12). A total of 14 (82%) patients were refractory to lenalidomide, seven (41%) patients refractory to pomalidomide, 12 (71%) patients refractory to bortezomib, six (35%) patients refractory to carfilzomib, and 12 (71%) patients dual refractory to both IMiDs and PIs. High‐risk FISH including del 17p, t(4;14), t(14;16), +1q21, and/or ‐1p were present in seven (50%) out of 14 patients with evaluable FISH data.

In the part one dose‐escalation phase, three patients each were treated at dose levels one, two, and three, and there were no DLTs. At dose level four, there were two DLTs among six patients treated including one patient with grade three sinusitis, and one patient who had a > 14 day delay to the start of cycle 2 day 1 due to grade three sinus tachycardia. Subsequently, two additional patients were enrolled at dose level three with no DLTs after which the study was prematurely closed to new patient enrollment due to other competing studies. Given that there were zero DLTs among five DLT‐evaluable patients treated at dose level three (and therefore a maximum potential <2 DLTs if all six patients had been enrolled to the cohort), dose level three (carfilzomib 20/36 mg/m^2^ on days 1, 2, 8, 9, 15, and 16, bendamustine 70 mg/m^2^ on days 1 and 2, and dexamethasone 20 mg on days 1, 2, 8, 9, 15, 16, 22, and 23) was determined to be the MTD.

The median number of cycles of treatment was 12 (range 2–64) among all 17 patients treated on study. The most common treatment emergent hematologic AEs of any grade included thrombocytopenia (76%), leukopenia (53%), anemia (47%), and neutropenia (29%) (Table S3). The most common grade 3/4 treatment emergent hematologic AEs were thrombocytopenia (24%), leukopenia (18%), and neutropenia (18%). The most common treatment emergent non‐hematologic AEs of any grade were cough (53%), fever (53%), and hyperglycemia (53%), and the most frequent treatment emergent grade 3/4 non‐hematologic AEs were any infection (53%) and fatigue (24%). Among AEs of special interest, peripheral neuropathy occurred in 29% of patients, including three grade two events and one grade three event, although all four of these patients had baseline grade one peripheral neuropathy. Heart failure occurred in one patient (grade three) at dose level three. The most common reason for treatment discontinuation was disease progression (N = 10), followed by adverse events (N = 3), which included grade three anaphylaxis to bendamustine at dose level two, grade three vomiting at dose level four, and grade three fatigue at dose level four. Other reasons for treatment discontinuation were withdrawal of consent (N = 2), lost to follow‐up (N = 1), and treating physician discretion (N = 1). There were no patient deaths during the study.

Among 17 response‐evaluable patients treated on study, the overall response rate (ORR, ≥ partial response) was 88% and ≥ very good partial response (VGPR) rate was 53% (Table 1). The clinical benefit rate (≥ minimal response) was 94%. The ORR at the MTD (dose level three) among five patients was 100% including four (80%) patients achieving a VGPR. Among 15 patients with ≥ PR, median duration of response was 14.8 months (95% CI 11.7, NA). Median PFS for all patients treated on study was 15.1 months (95% CI 11.1, NA) (Figure S1). Median PFS was 18.2 months (95% CI 12.7, NA) among six patients with 1–3 lines of prior therapy and 15.1 months (95% CI 4.8, NA) among 11 patients with >3 lines of prior therapy. Among 12 dual refractory patients, median PFS was 11.1 months (95% CI 5.8, NA). Among seven patients with high‐risk FISH, median PFS was 6.3 months (95% CI 2.7, NA) compared to 19.4 months (95% CI 17.1, NA) among seven patients without high‐risk FISH. Median OS for all patients treated on study was 56.3 months (95% CI 35.3, NA) at a median follow‐up of 56.7 months (Figure S2).

**TABLE 1:** Overall response

	All Dose Levels N = 17	Dose Levels 1 & 2 N = 6 Car 20/27 mg/m^2^ Benda 50–70 mg/m^2^	Dose Level 3 (MTD) N = 5 Car 20/36 mg/m^2^ Benda 70 mg/m^2^	Dose Level 4 N = 6 Car 20/45 mg/m^2^ Benda 70 mg/m^2^

sCR/CR, N (%)	1 (6%)	1 (17%)	0 (0%)	0 (0%)
VGPR, N (%)	8 (47%)	2 (33%)	4 (80%)	2 (33%)
PR, N (%)	6 (35%)	3 (50%)	1 (20%)	2 (33%)
MR, N (%)	1 (6%)	0 (0%)	0 (0%)	1 (17%)
ORR (≥PR), N (%)	15 (88%)	6 (100%)	5 (100%)	4 (67%)
CBR (≥MR), N (%)	16 (94%)	6 (100%)	5 (100%)	5 (83%)

Abbreviations: Benda, bendamustine; Car, carfilzomib; CBR, clinical benefit rate; CR, complete response; CR, stringent CR; MR, minimal response; MTD, maximum tolerated dose; ORR, overall response rate; PR, partial response; VGPR, very good partial response.

In this phase one study, we demonstrate the safety and preliminary efficacy of the combination of carfilzomib, bendamustine, and dexamethasone in RRMM. The MTD for this study was established at lower doses of carfilzomib and bendamustine compared to a recent phase one study evaluating this combination in newly diagnosed myeloma patients.^6^ In that study, the MTD was established at bendamustine 90 mg/m^2^ on days 1 and 2, carfilzomib 20/56 mg/m^2^ on days 1, 2, 8, 9, 15, and 16, and dexamethasone 20 mg on days 1, 2, 8, 9, 15, 16, 22, and 23 on a 28‐day cycle. The lower MTD doses of bendamustine (70 mg/m^2^) and carfilzomib (20/36 mg/m^2^) in this study is likely explained by the different study populations, as the current study enrolled a heavily pretreated RRMM population with a median of four lines of prior therapy.

The most common grade 3/4 AEs included thrombocytopenia, leukopenia, neutropenia, infection, and fatigue which was a predictable and manageable safety profile based on the known toxicities related to carfilzomib and bendamustine. Notably, four out of the nine grade three infections occurred among the six patients who received treatment at dose level four, which was one dose level higher than the final MTD. The ORR of 88%, ≥ VGPR rate of 53%, and median duration of response of 14.8 months suggests that this regimen has encouraging therapeutic activity in heavily pretreated patients with a median of four lines of prior therapy including dual‐refractory and carfilzomib‐refractory patients. Limitations of this study include the small number and heterogeneity of patients enrolled on study, the premature closure of the study which limited further safety and efficacy evaluation at the MTD, and the low number of patients who received prior mAb‐based therapy (one patient each was refractory to daratumumab and elotuzumab).

In summary, we establish the MTD of the combination of carfilzomib, bendamustine, and dexamethasone in RRMM and demonstrate its encouraging preliminary efficacy in this phase one study. These data suggest that carfilzomib in combination with the alkylating agent bendamustine may be a useful and relevant treatment option in this patient population.

## CONFLICT OF INTEREST

H.C.L. declares consulting fees from Amgen, Celgene, Genentech, GlaxoKlineSmith, Janssen, Sanofi, and Takeda and research funding from Amgen, Celgene, Daiichi Sankyo, GlaxoKlineSmith, Janssen, Regeneron, and Takeda.

E.E.M. declares consulting fees from Takeda, Celgene, Sanofi, GlaxoKlineSmith, and Adaptive Biotechnologies and research funding from Sanofi, Quest Diagnostics, Novartis, JW Pharma, and Merck.

R.Z.O. declares laboratory research funding from BioTheryX, and clinical research funding from CARsgen Therapeutics, Celgene, Exelixis, Janssen Biotech, Sanofi‐Aventis, Takeda Pharmaceuticals North America, Inc. Also, RZO has served on advisory boards for Amgen, Inc., Bristol‐Myers Squibb, Celgene, EcoR1 Capital LLC, Forma Therapeutics, Genzyme, GSK Biologicals, Ionis Pharmaceuticals, Inc., Janssen Biotech, Juno Therapeutics, Kite Pharma, Legend Biotech USA, Molecular Partners, Regeneron Pharmaceuticals, Inc., Sanofi‐Aventis, Servier, and Takeda Pharmaceuticals North America, Inc., and as a consultant for STATinMED Research. Finally, RZO is a Founder of Asylia Therapeutics, Inc., with associated patents and an equity interest, though this technology does not bear on the current manuscript.

M.L.W. declares consulting from Acerta Pharma, Janssen; Research Funding to UT MD Anderson from Acerta Pharma, Asana Biosciences, BeiGene, Celgene, Janssen, Juno Therapeutics, Kite Pharma, Onyx, Pharmacyclics, Proteolix; Honoraria from Celgene, Dava Oncology, Janssen, Proteolix,; Membership on an entity's Board of Directors or advisory committees: Janssen.

L.F., O.O., V.G., W.C., M.B., R.L., H.J.L, and P.J. reports no conflicts of interest.

## AUTHOR CONTRIBUTIONS

H.C.L. collected data, analyzed data, treated patients, and wrote the manuscript. L.F. performed biostatistical analysis. O.O., W.C., H.J.L., E.E.M., and R.Z.O treated patients. O.O., V.G., M.B., R.L. collected data. P.J. provided manuscript comments. M.L.W. designed the research, treated patients, and provided manuscript comments. All authors reviewed and approved the manuscript.

## Supporting information


**Appendix S1.** Supporting information

## Data Availability

The data that support the findings of this study are available from the corresponding author upon reasonable request.
