# Supplementary material for: A phase one trial of carfilzomib, bendamustine, and dexamethasone in relapsed and/or refractory multiple myeloma
Source: Am J Hematol. 2021 May 6;96(7):E243–6. doi: 10.1002/ajh.26178 (PMC8252413; doi:10.1002/ajh.26178)
Supplement: Supplementary file 1 — Appendix S1. Supporting information [file AJH-96-E243-s001.docx]

| **SUPPLEMENTARY TABLE 1. Phase 1 Dose Levels and Schedule (28-day cycle)** | | | |
| --- | --- | --- | --- |
|  |  |  |  |
|  | **Carfilzomib* mg/m2 IV Days 1, 2, 8, 9, 15, and 16** | **Bendamustine mg/m2 IV Days 1 and 2** | **Dexamethasone mg PO/IV Days 1, 2, 8, 9, 15, 16, 22, and 23** |
| **Dose Level -1** | 20 | 50 | 20 |
| **Dose Level 1** | 20/27 | 50 | 20 |
| **Dose Level 2** | 20/27 | 70 | 20 |
| **Dose Level 3** | 20/36 | 70 | 20 |
| **Dose Level 4** | 20/45 | 70 | 20 |
| **Dose Level 5** | 20/56 | 70 | 20 |
| **Dose Level 6** | 20/56 | 90 | 20 |
| *carfilzomib dosed at 20 mg/m2 on cycle 1, day 1 and 2. All subsequent infusions at the higher dose (as assigned in each cohort). | | | |
| For cycles 4-12, carfilzomib days 1, 2, 8, 9, 15, and 16; bendamustine day 1; dexamethasone on days 1, 2, 15, and 16. | | | |
| For cycles 13+, carfilzomib days 1, 2, 15, and 16; bendamustine day 1; dexamethasone on days 1 and 2 | | | |

# SUPPLEMENTARY TABLE 2. Baseline Patient Characteristics

| **Patients Enrolled, N** | 17 | |  |  |  |  |
| --- | --- | --- | --- | --- | --- | --- |
| **Median Age, (range)** | 63 | | (56-78) |  |  |  |
| **Sex** |  | |  |  |  |  |
| **Male, N (%)** | | 12 | (71) |  |  |  |
| **Female, N (%)** | 5 | | (29) |  |  |  |
| **Median lines of therapy, (range)** | 4 | | (1-12) |  |  |  |
| **1-3 line, N (%)** | 6 | | (35) |  |  |  |
| **3+ lines, N (%)** | 11 | | (65) |  |  |  |
| **Previous Therapy** |  | |  |  |  |  |
| **Prior Lenalidomide, N (%)** | 16 | | (94) |  |  |  |
| **Prior Pomalidomide, N (%)** | 9 | | (53) |  |  |  |
| **Prior Bortezomib, N (%)** | 16 | | (94) |  |  |  |
| **Prior Carfilzomib, N (%)** | 8 | | (47) |  |  |  |
| **Refractory to Lenalidomide, N (%)** | 14 | | (82) |  |  |  |
| **Refractory to Pomalidomide, N (%)** | 7 | | (41) |  |  |  |
| **Refractory to Bortezomib, N (%)** | 12 | | (71) |  |  |  |
| **Refractory to Carfilzomib, N (%)** | 6 | | (35) |  |  |  |
| **Refractory to IMiD and Proteasome Inhibitor, N (%)** | 12 | | (71) |  |  |  |
| **Prior autologous stem cell transplantation** | 15 | | (88) |  |  |  |
| **High risk FISH (del 17p, t(4;14) and/or t(14;16)), N (%)*** | 1 | | (8) |  |  |  |
| **High risk FISH (del 17p, t(4;14), t(14;16), +1q21, and/or -1p), N (%)°** | 7 | | (50) |  |  |  |
| *Among 13 evaluable patients | | | | |  |  |
| °Among 14 evaluable patients | | | | |  |  |

| **SUPPLEMENTARY TABLE 3. Treatment Emergent Adverse Events** | | | | |  |  |  | |  | |  | |  | |  | |  | |  | |  |
| --- | --- | --- | --- | --- | --- | --- | --- | --- | --- | --- | --- | --- | --- | --- | --- | --- | --- | --- | --- | --- | --- |
|  |  |  |  |  |  |  |  | |  | |  | |  | |  | |  | |  | |  |
|  | **Dose Levels 1 & 2, N=6** | | | | **Dose Level 3 (MTD), N=5** | | | | | | **Dose Level 4, N=6** | | | | | | | | **All Doses Levels, N=17** | |  |
|  | **Car 20/27 mg/m^2^, Benda 50-70 mg/m^2^** | | | | **Car 20/36 mg/m^2^, Benda 70 mg/m^2^** | | | | | | **Car 20/45 mg/m^2^, Benda 70 mg/m^2^** | | | | | | | |  |  |  |
| **Hematologic Adverse Events*** | **Grade 1 N, (%)** | **Grade 2 N, (%)** | **Grade 3 N, (%)** | **Grade 4 N, (%)** | **Grade 1 N, (%)** | **Grade 2 N, (%)** | **Grade 3 N, (%)** | | **Grade 4 N, (%)** | | **Grade 1 N, (%)** | | **Grade 2 N, (%)** | | **Grade 3 N, (%)** | | **Grade 4 N, (%)** | | **Total N (%)** | |  |
| Anemia | 1 (17%) | 1 (17%) | 1 (17%) |  |  | 2 (40%) |  | |  | |  | | 2 (33%) | | 1 (17%) | |  | | 8 (47%) | |  |
| Neutrophil count decreased |  | 1 (17%) | 1 (17%) |  |  | 1 (20%) |  | | 1 (20%) | |  | |  | | 1 (17%) | |  | | 5 (29%) | |  |
| Platelet count decreased | 1 (17%) | 1 (17%) | 1 (17%) |  | 2 (40%) | 1 (20%) |  | | 1 (20%) | | 1 (17%) | | 3 (50%) | | 2 (33%) | |  | | 13 (76%) | |  |
| White blood cell decreased | 3 (50%) | 1 (17%) | 1 (17%) |  |  | 1 (20%) | 1 (20%) | |  | |  | | 1 (17%) | | 1 (17%) | |  | | 9 (53%) | |  |
| **Non-Hematologic Adverse Events*** | **Grade 1 N, (%)** | **Grade 1 N, (%)** | **Grade 3 N, (%)** | **Grade 4 N, (%)** | **Grade 1 N, (%)** | **Grade 1 N, (%)** | **Grade 3 N, (%)** | | **Grade 4 N, (%)** | | **Grade 1 N, (%)** | | **Grade 1 N, (%)** | | **Grade 3 N, (%)** | | **Grade 4 N, (%)** | | **Total N (%)** | |  |
| Abdominal pain | 1 (17%) |  |  |  | 1 (20%) |  |  | |  | | 1 (17%) | |  | |  | |  | | 3 (18%) | |  |
| Allergic rhinitis | 2 (33%) |  |  |  | 2 (40%) |  |  | |  | | 4 (67%) | |  | |  | |  | | 8 (47%) | |  |
| Anaphylaxis |  |  |  |  |  |  |  | |  | |  | |  | | 1 (17%) | |  | | 1 (6%) | |  |
| Back pain | 1 (17%) |  |  |  |  | 1 (20%) |  | |  | |  | | 2 (33%) | |  | |  | | 4 (24%) | |  |
| Blurred vision | 2 (33%) | 1 (17%) |  |  | 2 (40%) |  |  | |  | | 5 (83%) | |  | |  | |  | | 10 (59%) | |  |
| Bronchial infection |  | 1 (17%) |  |  |  |  | 1 (20%) | |  | |  | |  | |  | |  | | 2 (12%) | |  |
| Bruising | 3 (50%) |  |  |  | 1 (20%) |  |  | |  | |  | |  | |  | |  | | 4 (24%) | |  |
| Chest pain |  |  |  |  | 1 (20%) |  |  | |  | | 3 (50%) | |  | |  | |  | | 4 (24%) | |  |
| Chills | 1 (17%) |  |  |  |  |  |  | |  | | 4 (67%) | |  | |  | |  | | 5 (29%) | |  |
| Constipation | 3 (50%) | 1 (17%) |  |  | 1 (20%) |  |  | |  | | 5 (83%) | | 1 (17%) | |  | |  | | 11 (65%) | |  |
| Cough | 4 (67%) |  |  |  | 1 (20%) | 1 (20%) |  | |  | | 3 (50%) | |  | |  | |  | | 9 (53%) | |  |
| Creatinine increased | 2 (33%) |  |  |  | 1 (20%) |  |  | |  | |  | |  | |  | |  | | 3 (18%) | |  |
| Diarrhea | 3 (50%) | 1 (17%) |  |  | 1 (20%) |  |  | |  | | 4 (67%) | | 1 (17%) | |  | |  | | 10 (59%) | |  |
| Dizziness | 4 (67%) |  |  |  | 1 (20%) |  |  | |  | | 3 (50%) | | 2 (33%) | |  | |  | | 10 (59%) | |  |
| Dry eye |  |  |  |  |  |  |  | |  | | 3 (50%) | |  | |  | |  | | 3 (18%) | |  |
| Dyspnea | 4 (67%) | 1 (17%) |  |  | 1 (20%) | 2 (40%) |  | |  | | 1 (17%) | | 2 (33%) | | 1 (17%) | |  | | 12 (71%) | |  |
| Edema limbs | 1 (17%) |  |  |  |  | 1 (20%) |  | |  | | 1 (17%) | |  | |  | |  | | 3 (18%) | |  |
| Fatigue | 1 (17%) | 3 (50%) | 1 (17%) |  | 2 (40%) | 1 (20%) | 1 (20%) | |  | | 2 (33%) | | 2 (33%) | | 1 (17%) | |  | | 14 (82%) | |  |
| Fever | 2 (33%) |  |  |  | 1 (20%) |  |  | |  | | 5 (83%) | |  | | 1 (17%) | |  | | 9 (53%) | |  |
| Generalized muscle weakness | 2 (33%) |  |  |  |  |  |  | |  | |  | |  | |  | |  | | 2 (12%) | |  |
| Headache | 1 (17%) | 1 (17%) |  |  |  |  |  | |  | | 2 (33%) | | 2 (33%) | |  | |  | | 6 (35%) | |  |
| Heart failure |  |  |  |  |  |  | 1 (20%) | |  | |  | |  | |  | |  | | 1 (6%) | |  |
| Hyperglycemia | 2 (33%) | 1 (17%) | 1 (17%) |  |  | 1 (20%) |  | | 1 (20%) | | 1 (17%) | | 2 (33%) | |  | |  | | 9 (53%) | |  |
| Hypertension |  |  | 1 (17%) |  | 1 (20%) |  |  | |  | |  | | 1 (17%) | | 1 (17%) | |  | | 4 (24%) | |  |
| Hyperuricemia |  |  |  |  | 1 (20%) |  |  | |  | | 2 (33%) | |  | |  | |  | | 3 (18%) | |  |
| Hypocalcemia |  |  |  |  |  |  |  | |  | |  | |  | |  | | 1 (17%) | | 1 (6%) | |  |
| Hypokalemia |  |  |  |  |  |  |  | |  | | 1 (17%) | |  | | 1 (17%) | |  | | 2 (12%) | |  |
| Hypomagnesemia | 1 (17%) |  |  |  |  |  |  | |  | | 4 (67%) | |  | |  | |  | | 5 (29%) | |  |
| Hypotension |  |  |  |  |  |  |  | |  | |  | |  | | 1 (17%) | |  | | 1 (6%) | |  |
| Infections and infestations |  | 1 (17%) | 1 (17%) |  |  | 1 (20%) | 1 (20%) | |  | | 1 (17%) | |  | | 1 (17%) | |  | | 6 (35%) | |  |
| Insomnia |  |  |  |  | 1 (20%) |  |  | |  | | 1 (17%) | | 1 (17%) | |  | |  | | 3 (18%) | |  |
| Lung infection |  |  |  |  |  |  |  | |  | |  | |  | | 1 (17%) | |  | | 1 (6%) | |  |
| Memory impairment | 2 (33%) |  |  |  | 2 (40%) |  |  | |  | | 1 (17%) | |  | |  | |  | | 5 (29%) | |  |
| Mucositis oral | 1 (17%) | 1 (17%) |  |  |  | 2 (40%) |  | |  | | 2 (33%) | | 1 (17%) | |  | |  | | 7 (41%) | |  |
| Myalgia | 3 (50%) | 1 (17%) |  |  | 2 (40%) | 1 (20%) |  | |  | | 2 (33%) | | 2 (33%) | |  | |  | | 11 (65%) | |  |
| Nasal congestion | 1 (17%) | 1 (17%) |  |  |  |  |  | |  | | 1 (17%) | |  | |  | |  | | 3 (18%) | |  |
| Nausea | 3 (50%) | 2 (33%) |  |  |  | 2 (40%) |  | |  | | 3 (50%) | | 2 (33%) | |  | |  | | 12 (71%) | |  |
| Pain | 1 (17%) | 1 (17%) |  |  | 1 (20%) |  | 1 (20%) | |  | |  | |  | |  | |  | | 4 (24%) | |  |
| Pain in extremity | 1 (17%) | 1 (17%) |  |  | 1 (20%) |  |  | |  | | 1 (17%) | | 2 (33%) | |  | |  | | 6 (35%) | |  |
| Palpitations |  | 1 (17%) |  |  |  | 1 (20%) |  | |  | | 2 (33%) | |  | |  | |  | | 4 (24%) | |  |
| Peripheral sensory neuropathy |  | 3 (50%) |  |  |  |  |  | |  | | 1 (17%) | |  | | 1 (17%) | |  | | 5 (29%) | |  |
| Rectal hemorrhage |  |  |  |  |  |  | 1 (20%) | |  | |  | |  | |  | |  | | 1 (6%) | |  |
| Sinus tachycardia |  |  |  |  |  |  |  | |  | | 1 (17%) | |  | | 1 (17%) | |  | | 2 (12%) | |  |
| Sinusitis |  | 1 (17%) |  |  |  |  |  | |  | |  | | 1 (17%) | | 1 (17%) | |  | | 3 (18%) | |  |
| Skin and subcutaneous tissue disorders | 1 (17%) |  |  |  | 1 (20%) |  |  | |  | | 3 (50%) | |  | |  | |  | | 5 (29%) | |  |
| Sore throat | 1 (17%) |  |  |  |  |  |  | |  | | 2 (33%) | |  | |  | |  | | 3 (18%) | |  |
| Surgical and medical procedures |  | 2 (33%) |  |  |  |  |  | |  | |  | | 1 (17%) | |  | |  | | 3 (18%) | |  |
| Upper respiratory infection |  | 3 (50%) | 1 (17%) |  |  | 2 (40%) |  | |  | |  | | 3 (50%) | | 2 (33%) | |  | | 11 (65%) | |  |
| Urinary tract infection |  |  | 1 (17%) |  |  |  |  | |  | |  | |  | |  | |  | | 1 (6%) | |  |
| Vomiting | 1 (17%) | 1 (17%) |  |  | 1 (20%) | 1 (20%) |  | |  | | 1 (17%) | | 1 (17%) | | 1 (17%) | |  | | 7 (41%) | |  |
| Watering eyes |  |  |  |  | 1 (20%) |  |  | |  | | 2 (33%) | | 1 (17%) | |  | |  | | 4 (24%) | |  |
| *AEs occurring in at least 15% of patients and all grade 3 and 4 AEs, regardless of attribution | | | | | | | |  | |  | |  | |  | |  | |  | |  | |

| **SUPPLEMENTARY FIGURE 1. Progression Free Survival of all Patients Enrolled on Study (N=17)**  ****  **SUPPLEMENTARY FIGURE 2. Overall Survival of all Patients Enrolled on Study (N=17)**  **** |
| --- |
